# Randomized controlled trials comparing gastric bypass, gastric band, and sleeve gastrectomy: A systematic review examining validity and applicability to wider clinical practice

**DOI:** 10.1111/obr.13718

**Published:** 2024-02-12

**Authors:** Katy A. Chalmers, Sian E. Cousins, Jane M. Blazeby, the By‐Band‐Sleeve Trial Management Group

**Affiliations:** ^1^ National Institute of Health and Care Research (NIHR) Biomedical Research Centre at University Hospitals Bristol NHS Foundation Trust and the University of Bristol, Surgical Innovation Theme and the Medical Research Council ConDuCT‐II Hub for Trials Methodology Research, Bristol Centre for Surgical Research, Population Health Sciences, Bristol Medical School Bristol UK

**Keywords:** applicability, bariatric surgery, PRECIS‐2, randomized controlled trials, validity

## Abstract

Consideration of how applicable the results of surgical trials are to clinical practice is important to inform decision‐making. Randomized controlled trials comparing at least two surgical interventions (of gastric bypass, gastric band, and sleeve gastrectomy) for severe and complex obesity were examined using the PRagmatic Explanatory Continuum Indicator Summary‐2 tool, to consider how applicable the trial results are to clinical practice, and the Risk of Bias 2 tool, to examine validity. MEDLINE, Embase, and CENTRAL databases were searched for studies published between November 2013 and June 2021, and 15 were identified. Using the PRagmatic Explanatory Continuum Indicator Summary‐2 tool, three were classified as pragmatic, with good applicability to clinical practice. Ten had more explanatory domains but did include some pragmatic characteristics, and two were predominantly explanatory. This was due to some trial design features that would not be considered applicable to the wider clinical setting, including being single‐centered, having prescribed intervention delivery methods, and intensive follow‐up regimens. Only two trials had low risk of bias, of which one was considered pragmatic. Three had high risk of bias. Overall, few trials in bariatric surgery are pragmatic with low risk of bias. Well‐designed pragmatic trials are needed to inform practice and reduce research waste.

**List of Abbreviations**
PRECIS‐2: PRagmatic Explanatory Continuum Indicator Summary‐2
RCT: randomized controlled trial
RYGB: Roux‐en‐Y gastric bypass

## 1 INTRODUCTION

Obesity is a complex disease, associated with a myriad of comorbidities including cardiovascular disease, type 2 diabetes, dyslipidemia, and depression, which negatively affects quality of life and is associated with premature death.^1^ In the United Kingdom, 28% of adults have obesity and 3.5% have severe and complex obesity^2^ (i.e., BMI ≥ 40 kg/m^2^ or a BMI ≥ 35 kg/m^2^ with obesity‐related comorbidities^3^; WHO class III and class II criteria, respectively).^4^ First‐line treatments involve modifications to diet, physical activity levels, and behavior and pharmacological interventions.^5^ Bariatric surgery is recommended only to selected individuals fulfilling specific criteria.^5^ Data suggest that, before COVID‐19, the number of procedures performed annually was increasing. Audit reports show that in 2015, the United Kingdom and United States performed 6177 and 196,700 surgical procedures, respectively, increasing to 7017 and 252,564 in 2018.^6^, ^7^ In the last decade, the Roux‐en‐Y gastric bypass (RYGB) has been the dominant procedure, overtaking gastric band.^6^ However, more recently, the number of sleeve gastrectomy procedures has surpassed RYGB, representing 43–70% of all bariatric surgeries performed in European countries^8^ and 59% in the United States.^7^ The National Institute for Health and Care Excellence currently does not recommend any specific bariatric surgical procedure but advises that surgeon–patient consultations should include information about surgeons' experience and the best available evidence on effectiveness and long‐term effects of procedures to inform decisions.^5^ When considering evidence from randomized controlled trials (RCTs), the validity and applicability of a study are critical in determining the relevance of the evidence to the wider clinical setting.

Since 2019, 10 systematic reviews have examined RCTs evaluating bariatric procedures, including RYGB, gastric band, and sleeve gastrectomy,^9^, ^10^, ^11^, ^12^, ^13^, ^14^, ^15^, ^16^, ^17^, ^18^ and the most recent review included studies published up to 2021.^12^ Five of the reviews were selective in the RCTs they included; two excluded studies that recruited patients with type 2 diabetes^9^, ^11^; and three excluded studies that did not measure specific outcomes of interest at 5‐year follow‐up.^10^, ^15^, ^16^ Six^9^, ^11^, ^13^, ^14^, ^17^, ^18^ of the 10 reviews assessed study validity, that is, the extent to which the observed treatment effect may be due to bias.^19^ However, only two^12^, ^16^ of these used the most recent tool for assessing validity (Cochrane's Risk of Bias v2 tool).^20^ None of the reviews discussed how applicable the trial results were to the wider clinical setting (i.e., pragmatic they were). This is crucial because it provides a guide about the extent to which information from the study is applicable to clinical practice across a range of settings. Key trial features such as eligibility criteria, number of centers, standardization of intervention, and follow‐up schedule affect the degree to which the trial may be considered pragmatic. For example, single‐center studies mean that interventions may be performed by the same small, highly experienced surgical team or that patients may come from a similar cultural background, both which may not be reflective of hospitals and populations elsewhere. Delivering trials under ideal conditions that do not reflect wider clinical practice (as in explanatory trials) would provide an understanding of how treatments work but would limit the degree to which results may be applied to wider clinical practice. The GRADE framework^21^, which was applied by three systematic reviews^9^, ^12^, ^13^, asks users to consider different factors that could strengthen or weaken the quality of the evidence. One of these factors is “indirectness” (applicability) and addresses the relevance of the population, intervention, comparator, and outcome of the published study to the population of interest^22^. While this considers some important aspects of trial design that influence the applicability of a trial, the PRagmatic Explanatory Continuum Indicator Summary‐2 (PRECIS‐2) tool^23^ goes further in this specific domain. Developed to guide trialists in designing a trial as they intended (i.e., explanatory or pragmatic), PRECIS‐2 considers nine key trial characteristics such as eligibility criteria, flexibility of delivery, and primary analysis.^23^ Awareness of the importance of applicability in trials is evidenced by the increase in the number of protocols citing PRECIS‐2 during trial design.^24^ The retrospective application of the tool further shows an interest by end users to evaluate the applicability of trial results from published studies^25^.

Given the current limitations of the synthesized evidence for RYGB, gastric band, and sleeve gastrectomy, the aim of this paper was to perform an up‐to‐date comprehensive review of the validity and applicability of RCTs in surgery for severe and complex obesity using up‐to‐date, validated tools.

## 2 METHODS

Annual literature searches were undertaken to systematically identify eligible RCTs. These were performed to inform the steering committee of an ongoing large RCT comparing RYGB, gastric band, and sleeve gastrectomy^26^, ^27^ of up‐to‐date evidence. The identified trials formed the data set for this systematic review. The findings are reported according to the updated Preferred Reporting Items for Systematic Reviews and Meta‐Analyses statement.^28^

### 2.1 Searches and screening

Six annual consecutive searches of electronic databases (Ovid MEDLINE, Ovid Embase, and CENTRAL) were conducted for RCTs published between (i) November 2013 and November 2016, (ii) November 2016 and October 2017, (iii) November 2017 and October 2018, (iv) October 2018 and October 2019; (v) October 2019 and October 2020, and (vi) November 2020 and June 2021. A comprehensive search strategy (Table S1) was developed, tailored to each database using the concepts of obesity, generic bariatric surgery, and individual surgical bariatric procedures. Ovid MEDLINE and Ovid Embase searches were filtered to include only RCTs; the CENTRAL database separates search results by study type, and therefore, an RCT filter was not required. Retrieved articles were imported into an Endnote database (version X9) and deduplicated. One assessor (K. A. C.) screened titles and abstracts, and ineligible articles were excluded. Full texts of remaining articles were retrieved and assessed for eligibility. If eligibility was unclear, a second reviewer (S. E. C.) was approached and, if necessary, a third senior reviewer (J. M. B.). Studies identified were presented to the By‐Band‐Sleeve study management group comprising bariatric surgeons, methodologists, and experts in obesity and bariatric surgery. This provided assurance that the searches and screening were comprehensive.

### 2.2 Study eligibility

Included studies were RCTs (including ancillary studies, substudies, and long‐term follow‐ups) comparing *at least* two of RYGB, gastric banding, *or* sleeve gastrectomy in people with severe and complex obesity, with or without type 2 diabetes. A study including any other intervention was only included if it also assessed at least two of the three target interventions. Protocols of included RCTs and clinical trial registry database entries were retrieved where available. Where protocols were not available, corresponding authors were contacted by email by the senior author (J. M. B.) and asked to provide a copy. Reviews, conference abstracts, feasibility and pilot studies, protocols, and those reporting only technical components of the procedures were excluded, as were articles not in the English language.

### 2.3 Data extraction

Data extraction focused on three main aspects: key trial characteristics; assessment of pragmaticism; and assessment of validity. Where multiple publications and/or protocols related to included RCTs, data were extracted for each trial, rather than each paper. Data were extracted by two researchers (K. A. C. and S. E. C.); a third reviewer (J. M. B.) was consulted where technical advice was required or if disagreements occurred. Key trial characteristics extracted included the surgical intervention (e.g., RYGB, band, or sleeve), number of centers, number of patients randomized, and study countries.

### 2.4 Assessment of pragmatism

The PRECIS‐2 tool, created to help trialists examine how trial design decisions can impact the applicability of the trial to usual care,^23^ was applied to all included RCTs by two reviewers (K. A. C. and S. E. C.) independently. Discussions with an experienced surgeon (J. M. B.) prior to assessments established parameters of usual care practice. Each trial was scored from 1 (*very explanatory*) to 5 (*very pragmatic*) for each of the nine domains (eligibility, recruitment, setting, organization, flexibility: delivery, flexibility: adherence, follow‐up, primary outcome, and primary analysis; see Table S2 for domain descriptions). When insufficient information was reported to complete the domain, it was left blank.^29^ Assessments were completed in small batches to compare ratings and discuss disagreements and enable standardization of judgments.

Specific aspects of PRECIS‐2 that required attention because of the nature of the interventions are explained here. The “eligibility” domain looks at the inclusion and exclusion criteria of the trial. In the context of bariatric surgery, exclusion of patients with gastroesophageal reflux disease was considered pragmatic in studies evaluating sleeve gastrectomy because sleeve gastrectomy would not be recommended for this patient group in the wider clinical practice. However, studies excluding certain patient groups (e.g., patients without type 2 diabetes) to examine specific outcomes, such as diabetes remission, were considered less pragmatic as additional study outcomes, such as weight loss, would not be applicable to the wider patient population eligible for bariatric surgery. “Flexibility: delivery” examines how much flexibility in the delivery of the intervention is allowed within the trial, for example, whether a strict prescribed protocol was utilized or whether delivery was based on surgeons' discretion. Judgments of procedural standardization were informed by deconstructing interventions using a validated typology^30^, ^31^ in consultation with a senior surgeon with knowledge of the three interventions (J. M. B.). “Flexibility: adherence” examines whether the intervention was delivered as intended and typically relates to a patient's compliance with the intervention (e.g., when taking medication). In surgical trials, PRECIS‐2 authors therefore suggest this domain should be left blank as it is not applicable.^23^ However, methods may be utilized within surgical trials to ensure that interventions are delivered as planned (e.g., by video recording procedures and capturing and analyzing data on compliance). This information can identify deviations from protocol and inform assessments for this domain. In line with PRECIS guidance, for example, trials that are very explanatory in nature may involve detailed protocols that require strict surgeon adherence to the intervention, whereas more widely applicable trials may have just a few components of the intervention to be delivered or prohibited. In either setting, intervention adherence can be assessed.

PRECIS wheels^23^ were created for each trial to illustrate assessments for each domain and were examined in conjunction with overall mean scores to provide a descriptive summary of domain assessments within and between trials. In the absence of a recognized standardized method for utilizing the scores to define trials as explanatory or pragmatic, in the current review, trials were considered pragmatic if they were conducted in multiple centers and had a mean score of 4 or above. Descriptive accounts of all domains are provided for each trial.

### 2.5 Assessment of validity

The Risk of Bias 2 tool^20^ was used to assess validity. Assessments were undertaken independently by two researchers with experience with the tool (K. A. C. and S. E. C.). The tool provides a framework for considering risk of bias in RCTs, enabling the user to evaluate the validity of the trial result of interest. The judgments of five domains (Table S3) are considered collectively to produce an overall risk of bias for the result for each outcome of interest (low risk, some concerns, or high risk). Each domain comprises several questions with five possible judgments—yes, probably yes, probably no, no, and no information. To aid the assessment process, the Risk of Bias 2 development group created a tool within Microsoft Excel™ in which judgments, informed by extensive tool guidance,^12^ could be inputted and the recommended overall risk of bias judgments computed for each trial. For this systematic review, the risk of bias was considered only for the primary outcome. Verbatim text from papers was extracted and used as a narrative to support judgments. Disagreements were resolved by consensus. Table S2 describes each domain and outlines relevant data to inform judgments for each.

## 3 RESULTS

### 3.1 Screening and included studies

**FIGURE 1:**
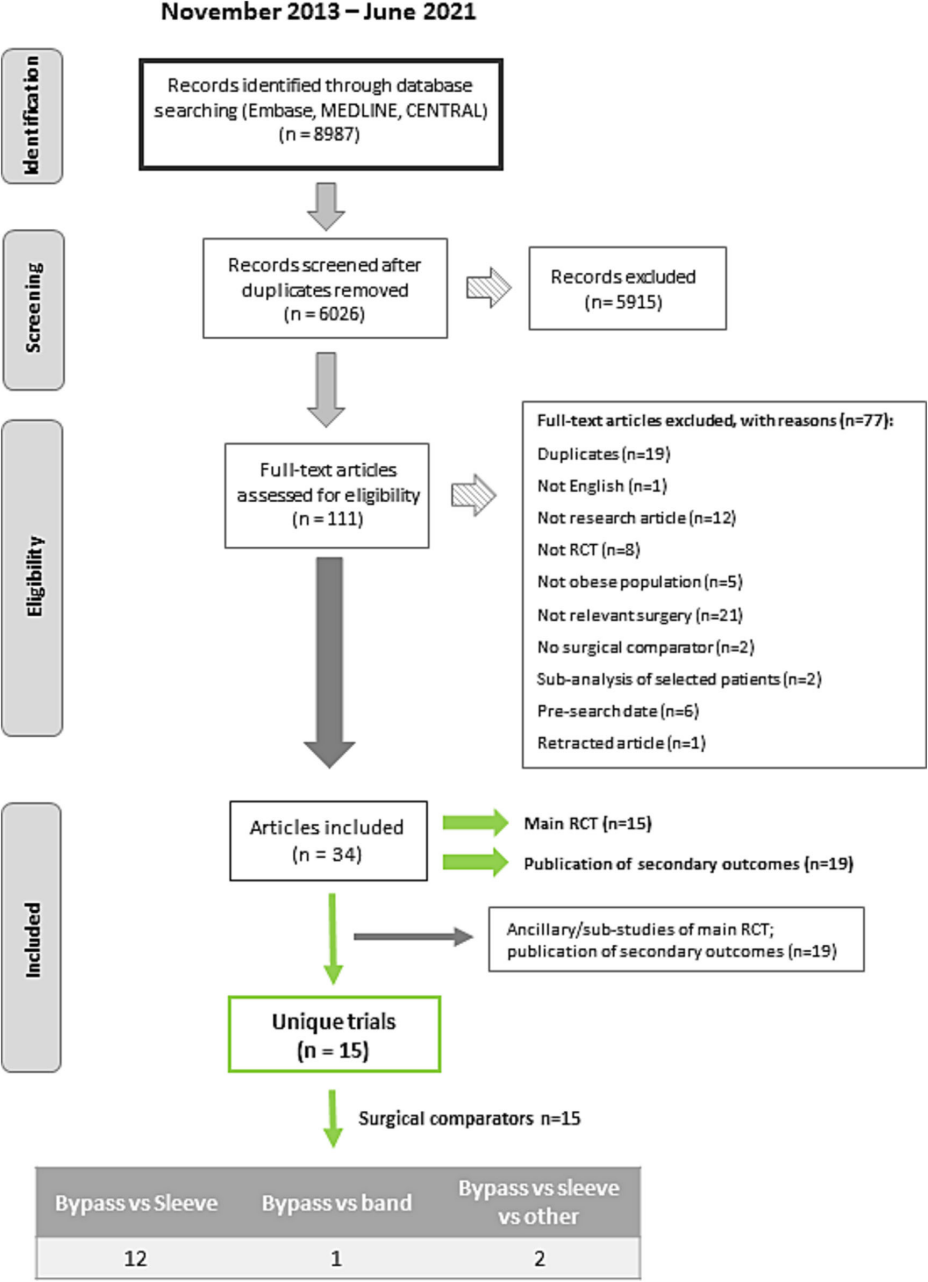
Preferred Reporting Items for Systematic Reviews and Meta‐Analyses flowchart depicting the search strategy and selection of articles for the review. RCT, randomized controlled trial.

Over the six annual review updates performed, 6026 titles and abstracts were screened. Full texts of 111 articles were reviewed, and 15 unique RCTs were included, based on information from 34 articles (Table S4).^32^, ^33^, ^34^, ^35^, ^36^, ^37^, ^38^, ^39^, ^40^, ^41^, ^42^, ^43^, ^44^, ^45^, ^46^, ^47^, ^48^, ^49^, ^50^, ^51^, ^52^, ^53^, ^54^, ^55^, ^56^, ^57^, ^58^, ^59^, ^60^, ^61^, ^62^, ^63^, ^64^, ^65^ Figure 1 summarizes this information.

### 3.2 Key trial characteristics

Study characteristics of the included trials are shown in Table 1. Details of ancillary, substudies, and follow‐up papers are shown in Table S4.

**TABLE 1:** Detailed characteristics of included studies.

Study number	First author^a^ (publication year)	Country	Number of centers	Excludes patients without T2D	Number of patients randomized			Primary outcome (timing of outcome	Trial name (trial registry number)
					RYGB	Sleeve	Other		
1	Keidar (2013)	Israel	1	Yes	20	21	—	HbA1c levels (1 year)	(NCT00667706)
2	Peterli (2018)^b^	Switzerland	4	No	112	113	—	Weight loss (5 years)	SM‐BOSS (NCT00356213)
3	Zhang (2014)	China	1	Yes	32	32	—	Weight loss (5 years)	—
4	Ignat (2017)^b^	France	1	No	45	55	—	Weight loss (5 years)	(NCT02475590)
5	De Barros (2020)^b^	Brazil	1	No	27	26	—	Evaluation of NAFLD (3 months)	(NCT02394353)
6	Kalinowski (2017)^b^	Poland	1	No	36	36	—	Weight loss (1 year)	(NCT01806506)
7	Biter (2020)^b^	Netherlands	1	No	316	321	—	Weight loss (2 years)	Sleeve Bypass (NL4573)
8	Casajoana (2017)^b^	Spain	1	Yes	15	15	15^c^	Markers of diabetes, weight loss, and body fat (1 year)	DIABETCIR (ISRCTN 14104758)
9	Nguyen (2017)	USA	1	No	125	—	125^d^	Weight loss (10 years)	(NCT00247377)
10	Schauer (2017)^b^	USA	1	Yes	50	50	50^e^	HbA1c levels (5 years)^f^	STAMPEDE (NCT00432809)
11	Capristo (2018)	Italy	1	Yes	60	60	—	Incidence of reactive hypoglycemia (1 year)	(NCT01581801)
12	Salminen (2018)^b^	Finland	3	No	119	121	—	Weight loss (5 years)	SLEEVEPASS (NCT00793143)
13	Hofsø (2020)^b^	Norway	1	Yes	54	55	—	Diabetes remission (1 year)	Oseberg (NCT01778738)
14	Pajecki (2020)^b^	Brazil	1	Yes	18	18	—	Surgical complications (30 days), mortality (90 days), and weight loss, improvements in diabetes, hypertension, and lipids (1 year)	BASE (NCT03339791)
15	Wallenius (2020)	Sweden	4	Yes	29	31	—	Diabetes remission (1 year)	CONTROL (NCT01984762)

Abbreviations: Hb1Ac, glycated hemoglobin; NAFLD, nonalcoholic fatty liver disease; RYGB, Roux‐en‐Y gastric bypass; T2D, type 2 diabetes.

^a^ If there is more than one follow‐up paper, the most recent publication is shown. If there are numerous ancillary/substudies associated with a study, the main paper is shown.

^b^ More than one publication associated with the study. See Table S1 for all publications identified in the review, including follow‐up, ancillary, and substudies.

^c^ Patients underwent greater curvature plication.

^d^ Patients had a gastric band fitted.

^e^ Patients received intensive medical therapy.

^f^ Primary outcome was measured at 12 months post‐randomization. Surgery performed as soon after randomization as possible.

Most trials (*n* = 13) compared two interventions; of these, 12 compared RYGB with sleeve gastrectomy and one compared RYGB with gastric band.^52^ Two trials compared three interventions, RYGB and sleeve gastrectomy with either intensive medical therapy^61^ or greater curvature plication.^36^ Eight trials excluded patients who did not have type 2 diabetes.^34^, ^36^, ^44^, ^49^, ^54^, ^61^, ^63^, ^64^ Eleven trials randomized fewer than 200 patients, including nine with 100 patients or fewer, and 11 recruited from a single center. Nine trials were conducted in nine different European countries, two trials in each of Brazil and United States, and one in each of Israel and China. None was international.

### 3.3 Assessment of pragmatism

**FIGURE 2:**
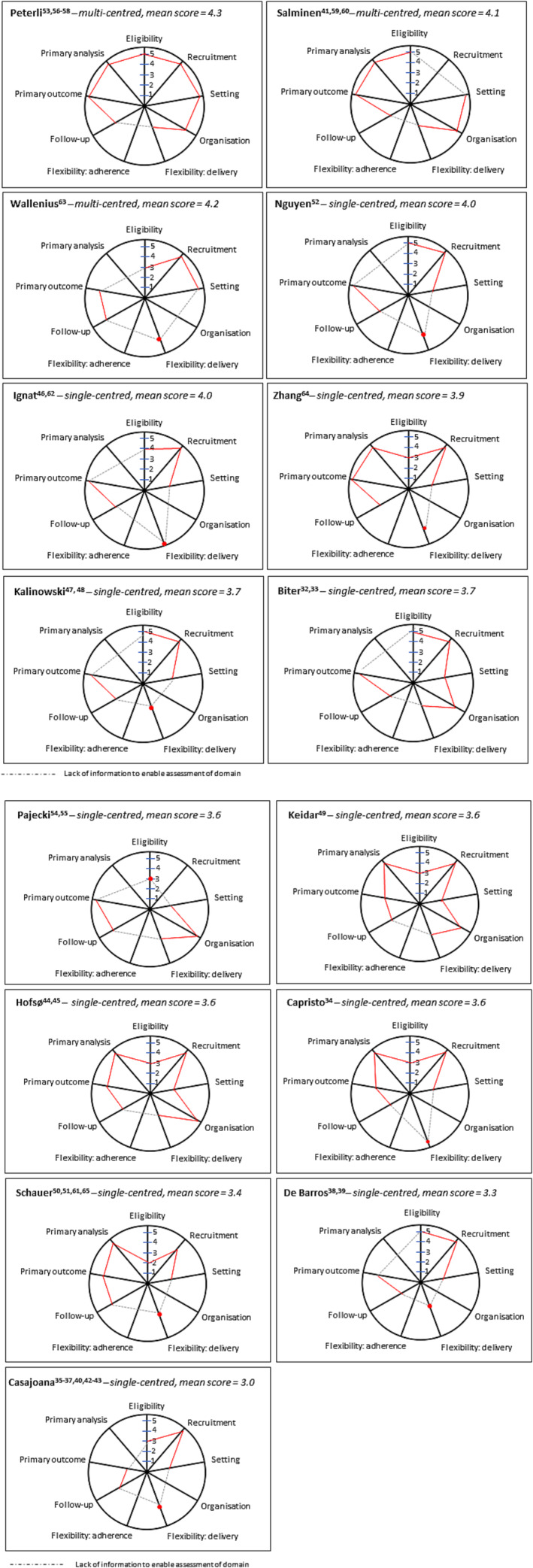
PRagmatic Explanatory Continuum Indicator Summary‐2 scores for each domain plotted on PRagmatic Explanatory Continuum Indicator Summary wheels, illustrating the pragmatism of included studies. Dashes (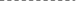) depict no information reported, therefore domain not assessed.

PRECIS‐2 assessments for the 15 studies are shown in Figure 2 and Table S5. Deviations from usual clinical practice, such as inclusion of patients with BMIs not usually considered eligible for surgery or additional scans to look for outcomes of interest, are observed as a score lower than 5. The greater the difference to usual practice, the lower the score. Assessment of all nine domains was not possible for any of the studies because key measures were not reported. None reported strategies to monitor the delivery of the interventions to ensure that they were performed as planned, and no information was included for the “recruitment” domain for two trials,^54^, ^60^ “organization” domain for nine trials,^34^, ^36^, ^39^, ^48^, ^52^, ^61^, ^62^, ^63^, ^64^ and “primary analyses” domain for five trials.^36^, ^38^, ^46^, ^47^, ^54^ Complete assessments of the remaining five domains (eligibility, setting, flexibility: delivery, follow‐up, and primary outcome) were possible for each study.

Three studies^56^, ^60^, ^63^ were considered to have a more pragmatic approach. In addition to being multicentered, these trials had pragmatic features including recruiting patients from outpatient clinics and evaluating a patient‐orientated primary outcome. However, even in these studies, restrictive eligibility criteria^63^, more intensive follow‐up regimens than usual care,^56^, ^60^ and standardization of interventions^56^, ^60^ were deemed more explanatory. Ten studies^33^, ^34^, ^44^, ^46^, ^48^, ^49^, ^52^, ^54^, ^61^, ^64^ had more explanatory domains including narrow eligibility criteria,^34^, ^44^, ^49^, ^54^, ^61^, ^64^ additional research‐specific follow‐up assessments and data collection,^33^, ^34^, ^44^, ^48^, ^49^, ^62^, ^64^ standardization of interventions,^33^, ^44^, ^48^, ^49^, ^54^, ^61^ and being conducted in one^34^, ^44^, ^48^, ^49^, ^54^, ^61^, ^62^, ^64^ or two^33^ centers. These trials also had some pragmatic characteristics, including recruitment from outpatient clinics and patient‐orientated primary outcomes. The remaining two studies^36^, ^39^ had mostly explanatory domains.

Most commonly, the “recruitment” domain was judged as pragmatic across studies, with patients recruited from outpatient clinics in 13 of the 15 studies.^33^, ^34^, ^36^, ^39^, ^44^, ^48^, ^49^, ^52^, ^56^, ^61^, ^62^, ^63^, ^64^ “Primary analyses” was reported by 10 of the 15 included studies,^33^, ^34^, ^44^, ^49^, ^52^, ^56^, ^60^, ^61^, ^63^, ^64^ seven of which used intention‐to‐treat analysis,^34^, ^44^, ^49^, ^56^, ^60^, ^61^, ^64^ ensuring patients were analyzed in their randomized group. Assessment of the “organization” domain was possible for six studies,^33^, ^44^, ^49^, ^54^, ^56^, ^60^ all rated as pragmatic. The lowest scoring domains, and therefore those judged to be more explanatory, were “trial setting,” “follow‐up,” and “flexibility: delivery.” Most studies were conducted in one (*n* = 11) or two (*n* = 1) centers, 12 studies^33^, ^34^, ^36^, ^39^, ^44^, ^48^, ^49^, ^52^, ^56^, ^60^, ^62^, ^64^ reported follow‐up assessment schedules that were more frequent and longer term that in usual care, and 10 studies^33^, ^36^, ^39^, ^44^, ^48^, ^49^, ^54^, ^56^, ^60^, ^61^ reported methods aimed at standardizing delivery of the interventions within the trial.

### 3.4 Assessment of validity

**FIGURE 3:**
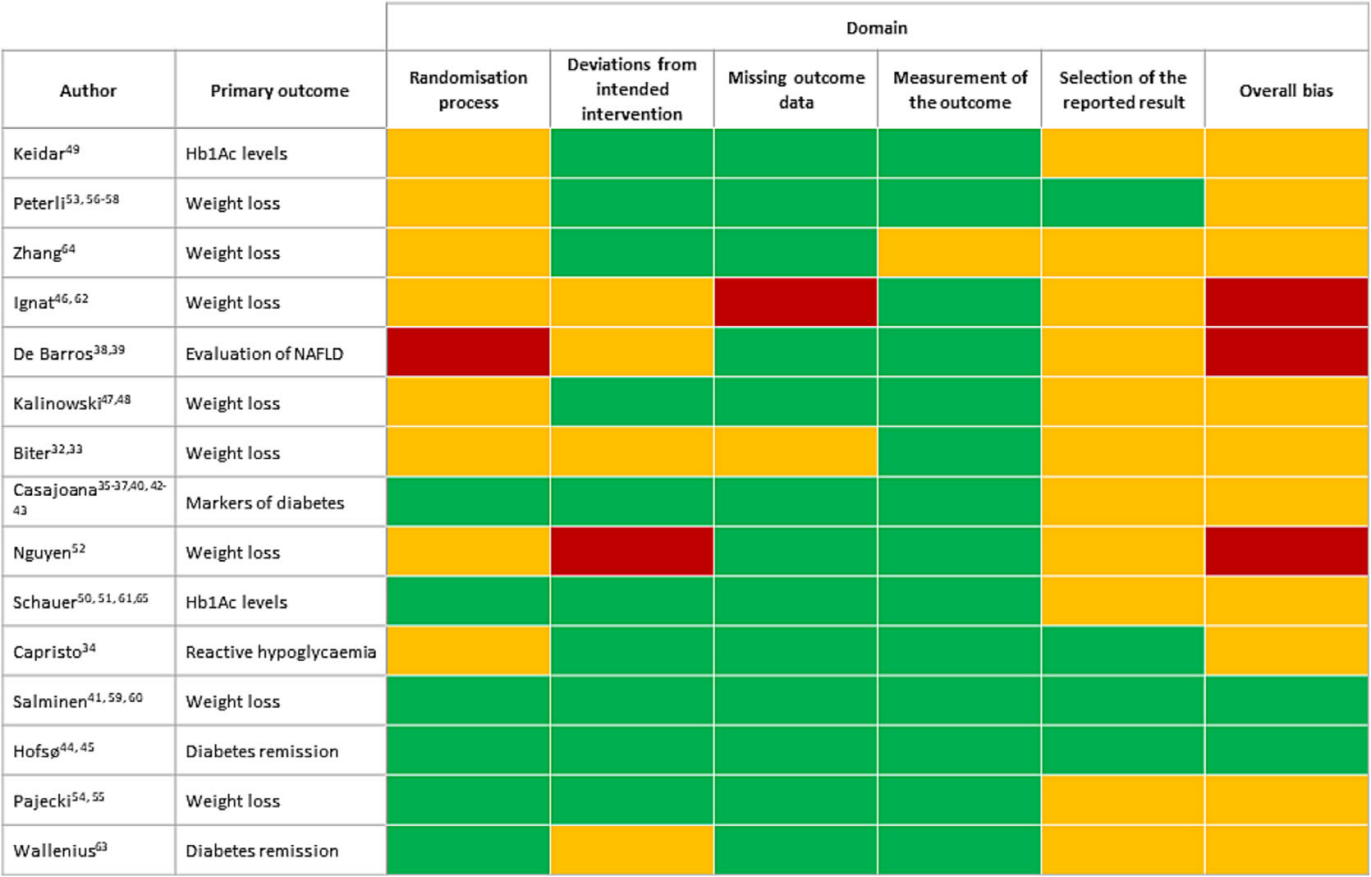
Risk of bias assessment for the primary outcome of each trial, using the Risk of Bias 2 tool. Color green illustrates low risk of bias (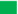), yellow some concerns (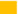), and red high risk of bias (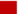).

Risk of bias judgments are illustrated in Figure 3 (see Table S6 for verbatim article text used to inform decisions).

Overall risk of bias was judged to be low in two studies^44^, ^60^ and high in three,^38^, ^46^, ^52^ with some concerns about the other 10. Of the 75 judgments made over the five domains, 47 (62.7%) were low risk of bias. Two domains were frequently judged to be at low risk of bias—“missing outcome data” and “measurement of outcome.” For the “missing outcome data” domain, 13^10^, ^34^, ^36^, ^44^, ^47^, ^49^, ^52^, ^54^, ^56^, ^60^, ^61^, ^63^, ^64^ of the 15 studies provided comprehensive reporting of data; of the remaining two studies, one was considered high risk of bias^62^ due to extensive loss of patients to follow‐up and the other, of “some concern,”^33^ with significant loss of patients to follow‐up but with single imputation for missing data performed. For the “measurement of outcome” domain, 14^33, 34, 36, 38, 44, 46, 47, 49, 52, 54, 56, 60, 61, 63^ of the 15 studies used methods deemed to be appropriate to assess primary outcomes, which were unlikely to be affected in the absence of outcome assessor blinding. The remaining study was judged to be of “some concern”^64^ because a notable number of patients did not attend clinic for assessment but measured their weight at home. In this circumstance, with the outcome assessors (patients) unblinded, there was a risk that the primary outcome (weight loss) could be influenced. In five studies, there were several reasons why “deviations from intended interventions” were not judged to be low risk. None used appropriate intention‐to‐treat analysis,^32^, ^33^, ^38^, ^39^, ^46^, ^52^, ^62^, ^63^ two excluded patients because insurance companies would not pay for procedures,^46^, ^52^ and two excluded patients who had a preference for the other procedure.^52^, ^63^ Six (40%) studies^36^, ^44^, ^54^, ^60^, ^61^, ^63^ comprehensively reported how randomization and allocation were performed.

## 4 DISCUSSION

This review provides an in‐depth examination of the applicability of RCTs evaluating three main bariatric procedures to wider clinical practice. This important area has received much less attention than assessment of study validity, which is commonly assessed with risk of bias tools.^19^, ^20^ The PRECIS‐2 tool is a well‐validated tool used for this purpose. In this review, assessment of all nine PRECIS‐2 domains was not achieved for any of the 15 included studies. This was due to poor reporting of key trial areas such as prerequisite surgical experience, assuring surgeons' adherence to protocol, and use of intention‐to‐treat analysis. Three studies^53^, ^56^, ^57^, ^58^, ^60^, ^63^ were classified as more pragmatic and therefore applicable to the wider clinical setting. The remaining studies were classified as either being “equally explanatory and pragmatic” (*n* = 10) or explanatory (*n* = 2). These studies had restrictive eligibility criteria, were conducted in one or two centers, prescribed intervention delivery methods, and an intense follow‐up regimen, which reduced their applicability to “real‐world” clinical practice. Two trials were at low risk of bias, one of which was considered pragmatic.^60^ There is a need for increased awareness of the importance of pragmatism within the surgical trials community to inform the design of high‐quality pragmatic, multicenter surgical RCTs that are needed for evidence‐based practice in this area.

The concept of a study being described as “pragmatic” or “explanatory” was first introduced in 1967.^66^ Trials that evaluated the intervention under conditions more aligned with usual care were described as having a pragmatic approach while trials assessing the intervention under idealized conditions explanatory.^66^ The pragmatism of a trial is closely associated with the applicability of a trial's results, in that the more the trial design resembles usual care, the more confidence there may be that trial results are applicable and can be implemented more widely. Only three studies examined were pragmatic. Eligibility criteria for trial entry is one parameter assessed. A pragmatic study would aim to include patients who would receive the intervention in usual care settings (i.e., have wide inclusion criteria).^3^, ^4^ Six studies^34^, ^36^, ^44^, ^49^, ^61^, ^63^ excluded patients without type 2 diabetes, and as such, the findings would not be applicable to a wider bariatric surgery population, only those with type 2 diabetes. The number of centers is also important to consider. Only three studies were conducted in more than two centers.^56^, ^60^, ^63^ In a single‐center study, the cultural and economic diversity of patients may be limited and hence less representative of the true bariatric population. Trials conducted in a specialist center with a highly experienced surgical team may not be comparable with clinical practice in hospitals that perform fewer procedures per year. Treatment effects have been found to be larger in single‐center studies,^67^, ^68^ and so, results should be interpreted with caution. It is only in multicenter trials that the intervention may be evaluated in different contexts with multiple surgeons, and this is more representative of what will happen when interventions take place outside the trial setting. While it is recognized that surgical trials may be more challenging than drug trials, multicenter surgical trials for bariatric surgery are feasible.^26^, ^56^, ^60^, ^63^ A domain that was found to be less pragmatic involved flexibility in the delivery of the interventions. More flexibility would suggest more pragmatism, and surgeons frequently undertake operations with variable components,^30^ such as limb length and pouch size.^69^, ^70^, ^71^ Five studies^33^, ^39^, ^44^, ^56^, ^60^ reported very detailed and standardized delivery protocols although fidelity to the intervention actually performed was not recorded. A typology to aid the identification of components of surgical interventions in trials and to aid delivery and quantify flexibility can be used to help with this challenge.^30^

Strengths of this review include rigorous and comprehensive assessment of methodological quality and applicability by two reviewers independently. The inclusion of detailed, verbatim text used to underpin judgments is provided in Supporting Information S1, ensuring transparency about how the tools were applied. A potential limitation to the review is that due to the nature of the annual reviews used to identify relevant trials, dual screening was not conducted. However, the findings from these reports were presented to experts involved in the By‐Band‐Sleeve study^26^, ^27^ to ensure comprehensiveness. The inclusion of trials investigating the gastric band may seem irrelevant to some readers given the reduction in its popularity among many bariatric surgeons. However, the procedure is still in use in some places and in private practice and was the third most common procedure at the outset of this review process. The decline in trials evaluating gastric band highlights and reflects the changes in clinical practice over the last decade. In the UK National Bariatric Surgical Registry, which included data from 2013 to 2018, 11% of all procedures were gastric band. It is considered that gastric band is still used in some places because of patient choice.^6^ The PRECIS‐2 tool used in this review was developed to aid trialists in the design stage rather than the assessment of completed studies. While the widely used GRADE framework considers the effects of some aspects of trial design on applicability in published studies^22^ and other tools have been developed to evaluate applicability specifically in systematic reviews,^72^ the validated PRECIS‐2 tool is increasingly being used retrospectively^25^ and the PRECIS‐2 authors concluded that it could be used retrospectively with some adjustments.^29^ Because of this endorsement and its ability to facilitate a more comprehensive and detailed evaluation, the current review utilized the PRECIS‐2 tool.

This review has demonstrated that trials in bariatric surgery published in the last 9 years contain characteristics that are both pragmatic and explanatory in nature but that only three could be assessed as fully pragmatic and thus have wide applicability. This lack of large, well‐designed, high‐quality RCTs may be due to the perceived difficulties in designing and running complex surgical RCTs^73^ but may also be attributed to the numerous national bariatric registries across the world, many of which are well‐organized and validated,^74^ which may render RCTs unnecessary. Indeed, data from over 300,000 procedures were collated by International Federation for the Surgery of Obesity and Metabolic disorders in 2022^74^—a large body of data from which to draw conclusions. However, while registries purportedly reflect real‐world evidence,^75^ there are inherent biases in normal clinical practice, whether it be a surgeon having a preferred procedure or a center having stringent inclusion criteria thus limiting the range of patients undergoing surgery. It remains, in our opinion, important to have both types of data and evidence. The strength of randomization to create groups without bias selection remains a powerful tool to compare and contrast surgical procedures. The methodological differences between the two have been written about in this important editorial.^75^

While the design and conduct of surgical RCTs may be more complex and hence require more thought, increased awareness of existing guidelines^76^, ^77^, ^78^, ^79^, ^80^ and tools^20^, ^23^ developed to improve design, conduct, and reporting of trials and consideration of how trial design characteristics impact the applicability of trial results to the wider clinical setting are central to improving study quality in this area, to inform clinical practice.

## AUTHOR CONTRIBUTIONS

J. M. B. conceived the idea for this study. It was designed by J. M. B., K. A. C., and S. E. C. J. M. B. designed the search strategy which was implemented by K. A. C. K. A. C. screened citations, and K. A. C. and S. E. C. performed data extraction. K. A. C. and S. E. C. applied the PRECIS‐2 and RoB 2 tools. J. M. B. acted as third reviewer if discrepancies arose. K. A. C. analyzed the data and constructed the tables and figures. J. M. B., K. A. C., and S. E. C. drafted the manuscript. J. M. B. provided content expertise and reviewed the manuscript critically for technical and methodological accuracy. The corresponding author attests that all listed authors meet authorship criteria and that no others meeting the criteria have been omitted. All authors reviewed and approved the final manuscript.

## CONFLICT OF INTEREST STATEMENT

J. M. B. is an NIHR Senior Investigator. The authors report no conflicts of interest.

## FUNDING INFORMATION

This study was supported by the MRC ConDuCT‐II (Collaboration and innovation for Difficult and Complex Randomized Controlled Trials in Invasive Procedures) Hub for Trials Methodology Research (MR/K025643/1) and NIHR (National Institute for Health and Care Research) Biomedical Research Centre at the University Hospitals Bristol NHS Foundation Trust and the University of Bristol (BRC‐1215‐20011). The By‐Band‐Sleeve study is funded by the National Institute for Health and Social Care Research Health Technology Assessment Programme (ref: 09/127/53). The views expressed in this publication are those of the authors and not necessarily those of the NHS or the Department of Health.

## Supporting information


**Table S1.** Search strategy for Ovid MEDLINE.
**Table S2.** Summary of details extracted from each RCT to inform judgments across the PRECIS‐2 domains^1^.
**Table S3.** Method for the assessment of internal validity using the risk of bias (ROB) 2 tool2.
**Table S4.** Descriptive details of included articles. Sub‐studies and ancillary studies are grouped with the original trial publication.
**Table S5.** Mean domain scores for PRECIS‐2 assessments.
**Table S6.** Verbatim text used to support risk of bias (ROB) judgment using the RoB 2 tool^2^ for included studies.

## Data Availability

Data supporting this study are included within the article and supporting materials.
