# Supplementary material for: Randomized controlled trials comparing gastric bypass, gastric band, and sleeve gastrectomy: A systematic review examining validity and applicability to wider clinical practice
Source: Obes Rev. 2024 Feb 12;25(5):e13718. doi: 10.1111/obr.13718 (PMC11478934; doi:10.1111/obr.13718)
Supplement: Supplementary file 1 — Table S1. Search strategy for Ovid MEDLINE. Table S2. Summary of details extracted from each RCT to inform judgments across the PRECIS‐2 domains1. Table S3. Method for the assessment of internal validity using the risk of bias (ROB) 2 tool2. Table S4. Descriptive details of included articles. Sub‐studies and ancillary studies are grouped with the original trial publication. Table S5. Mean domain scores for PRECIS‐2 assessments. Table S6. Verbatim text used to support risk of bias (ROB) judgment using the RoB 2 tool2 for included studies. [file OBR-25-e13718-s001.pdf]

Randomised controlled trials comparing gastric bypass, gastric band and sleeve gastrectomy: A systematic review examining validity and applicability to wider clinical practice.

Authors: Katy A Chalmers[1], Sian E Cousins[1] and Jane M Blazeby[1] on behalf of the By-Band-Sleeve Trial Management Group

[1] National Institute of Health and Care Research (NIHR) Biomedical Research Centre at University Hospitals Bristol NHS Foundation Trust and the University of Bristol, Surgical Innovation Theme and the Medical Research Council ConDuCT-II Hub for Trials Methodology Research, Bristol Centre for Surgical Research, Population Health Sciences, Bristol Medical School, Bristol, UK

Acknowledgements: With thanks to the By-Band-Sleeve Trial Management Group (TMG) for their support and for reviewing the manuscript. By-Band-Sleeve TMG: Rob C Andrews PhD, Medical Research, University of Exeter Medical School, Exeter, UK; John Bessent, Patient representative, UK; James P Byrne MD, University Hospital Southampton NHS Foundation Trust, Southampton, UK; Nicholas Carter MSc, Portsmouth Hospitals University NHS Trust, Portsmouth, UK; Caroline Clay†, Patient representative, UK; Jenny L Donovan PhD, Bristol Medical School, Population Health Sciences, University of Bristol, Bristol, UK; Eleanor Gidman PhD, Bristol Trials Centre, Bristol Medical School, University of Bristol, Bristol, UK; Graziella Mazza PhD, Bristol Trials Centre, Bristol Medical School, University of Bristol, Bristol, UK; Mary O'Kane MSc, Dietetic Department, Leeds Teaching Hospitals NHS Trust, Leeds, UK; Barnaby C Reeves DPhil, Bristol Trials Centre, Bristol Medical School, University of Bristol, Bristol, UK; Chris A Rogers PhD, Bristol Trials Centre, Bristol Medical School, University of Bristol, Bristol, UK; Nicki Salter DipHE, Somerset NHS Foundation Trust, Somerset, UK; Janice L Thompson PhD, School of Sport, Exercise & Rehabilitation Sciences, University of Birmingham, Birmingham, UK; Richard Welbourn MD, Somerset NHS Foundation Trust, Somerset, UK; Sarah Wordsworth PhD, Health Economics Research Centre, Nuffield Department of Population Health, University of Oxford, UK

†Passed away before reviewing and approving the final manuscript

Corresponding author:

Katy Chalmers

University of Bristol

Bristol Centre for Surgical Research

Canynge Hall, 39 Whatley Road

Bristol, BS8 2PS

United Kingdom

Email: katy.chalmers@bristol.ac.uk

Supplementary Table 1. Search strategy for Ovid MEDLINE

	**Obesity concept**
1	exp obesity/
2	Overweight/
3	over?weight.ti,ab.
4	over weight.ti,ab.
5	overeating.ti,ab.
6	over?eating.ti,ab.
7	exp Weight Loss/
8	weight loss.ti,ab.
9	weight reduc$.ti,ab
10	or/1-9
	**Bariatric surgery concept**
11	bariatric surg$.ti,ab
12	exp Bariatric Surgery/
13	(surg* adj5 bariatric).ti,ab.
14	anti?obesity surg$.ti,ab.
15	antiobesity surg$.ti,ab
16	(obesity adj5 surgery).ti,ab.
17	(obesity adj5 surgical).ti,ab.
18	(gastro?gastrostomy or "gastric bypass" or "gastric surgery" or "restrictive surgery").ti,ab
19	exp gastric Bypass/
20	gastrointestinal surg$.ti,ab.
21	gastrointestinal diversion$.ti,ab.
22	gastric band$.ti,ab.
23	silicon band*.ti,ab.
24	gastrectomy.ti,ab.
25	LAGB.ti,ab.
26	lap band$.ti,ab.
27	malabsorptive surg$.ti,ab.
28	"roux-en-Y".ti,ab.
29	Anastomosis, Roux-en-Y/
30	malabsorptive procedure$.ti,ab.
31	obesity/su
32	exp Obesity, Morbid/su [Surgery]
33	or/11-32
	**RCT concept**
34	exp randomized controlled trial/
35	randomi#ed controlled trial.tw
36	random*.tw
37	trial.tw
38	Random Allocation/

39 (random allocation).tw

40 or/34-39

Combination of concepts

41 10 AND 33 AND 40

Supplementary Table 2. Summary of details extracted from each RCT to inform judgments across the PRECIS-2 domains[1]

Domain	Description
**Eligibility**	Verbatim criteria used to include or exclude patients from the trials, grouped by theme where appropriate (e.g., assessing weight loss). Themes developed iteratively between the team.
**Recruitment**	Information regarding the type of centre (clinic, hospital department) where patients were recruited. Any strategies used to recruit patients to the trial, such as examining medical records or advertising.
**Setting**	Information regarding the setting of the trial, such as number, type and location of centres.
**Organisation**	Numbers of gastric band, gastric bypass and sleeve gastrectomy procedures performed by individual surgeons, participating centres, and surgical teams. Relevant text regarding level of experience required to take part in the trial, such as the number of procedures performed.
**Flexibility: delivery**	Any strategies that aimed to standardise surgical interventions, such as delivery of the interventions by the same surgeon/surgical team or prescriptive protocols.
**Flexibility: adherence**	Any strategies to ensure fidelity to the intervention, such as videorecording.
**Follow-up**	Details about the timing and frequency of follow-up visits, and additional data collected.
**Primary outcome**	Type of outcome, e.g., clinical or patient-reported, and its pertinence to patients.
**Primary analysis**	Ascertaining how data were analysed in the event of crossovers or protocol deviations, information regarding intention-to-treat (ITT) or per protocol analysis (or both).

Supplementary Table 3. Method for the assessment of internal validity using the risk of bias (ROB) 2 tool[2]

Domain	Description
**Randomisation process**	Information about the generation of the random sequence and allocation concealment were extracted, in addition to determination of any baseline difference between intervention groups.
**Deviation from intended interventions**	Reporting of blinding of participants and surgeons was extracted. Information referring to crossovers and protocol deviations was also extracted. Crossovers included both planned and unplanned crossovers between trial groups e.g., MIE to open or vice versa. Protocol deviations were defined as instances when patients either did not receive the intervention due to unforeseen circumstances (e.g., disease progression) or underwent an additional step or component not outlined in the protocol (e.g., additional resection due to suspicious findings).
**Missing outcome data**	Trial results were reviewed to assess the comprehensiveness of the data.
**Measurement of outcome data**	Primary outcomes were extracted, including measures used and timing of last measurement. If a primary outcome was not explicitly defined, the outcome used to calculate sample size was extracted, or if this was not available the first outcome reported in the abstract. If a composite outcome was reported (e.g., complications), all variables contributing to the composite outcome were extracted. Reporting of blinding of outcome assessors was extracted. If outcome assessors were not blinded, any likely influence on the assessment of the outcome was considered (e.g., patient-reported outcome or a blood test)
**Selection of reported results**	Protocols (where available) and pre-specified statistical plans were examined.

Supplementary Table 4. Descriptive details of included articles. Sub-studies and ancillary studies are grouped with the original trial publication.

Trial List ID	Author	Trial group 1	Trial group 2	Trial group 3	Primary outcome[1]	Timing of primary outcome (months)	Number of centres	Sample size[2]	Country
1[3]	Keidar (2013)	Bypass	Sleeve	-	Measure of diabetes	12	1	41	Israel
2a[4]	Peterli (2013)	Bypass	Sleeve	-	Measure of weight loss	12	4	217	Switzerland
2b[5]	Peterli (2017)	Bypass	Sleeve	-	Measure of weight loss	36	4	217	Switzerland
2c[6]	Peterli (2018)	Bypass	Sleeve	-	Measure of weight loss	60	4	217	Switzerland
2d	Nussbaumer (2020)	Bypass	Sleeve	-	Measure of glycaemic control	1	1	16	Switzerland
3	Zhang (2014)	Bypass	Sleeve	-	Measure of weight loss	60	1	64	China
4a[7]	Vix (2014)	Bypass	Sleeve	-	Measure of weight loss	12	1	100	France
4b	Ignat (2017)	Bypass	Sleeve	-	Measure of weight loss	60	1	100	France
5a[8]	De Barros (2015)	Bypass	Sleeve	-	Measure of diabetes	3	1	51	Brazil
5b	De Barros (2020)	Bypass	Sleeve	-	Measure of NAFLD	3	1	53	Brazil
6a[9]	Kalinowski (2017)	Bypass	Sleeve	-	Measure of NAFLD	12	1	72	Poland
6b[9]	Kalinowski (2017)	Bypass	Sleeve	-	Measure of gut hormones	12	1	72	Poland
7a[10]	Biter (2017)	Bypass	Sleeve	-	Measure of HRQL	12	1	150	Netherlands

7b[11]	Biter (2020)	Bypass	Sleeve	-	Measure of weight loss	24	2	637	Netherlands
8a	Casajoana (2017)	Bypass	Sleeve	GCP	Measure of gut hormones	12	1	45	Spain
8b[12]	Guerrero-Perez (2019)	Bypass	Sleeve	GCP	Measure of bone density	12	1	45	Spain
8c[12]	Ceperuelo-Mallafré (2019)	Bypass	Sleeve	GCP	Measure of diabetes	12	1	45	Spain
8d[13]	Ejarque (2019)	Bypass	Sleeve	GCP	Measure of diabetes	12	1	45	Spain
8e[12]	Guerrero-Perez (2020)	Bypass	Sleeve	GCP	Measure of bone density	60	1	45	Spain
8f[13]	Casajoana (2021)	Bypass	Sleeve	GCP	Measure of diabetes	60	1	45	Spain
9	Nguyen (2017)	Bypass	Band	-	Measure of weight loss	120	1	250	USA
10a	Schauer (2017)	Bypass	Sleeve	Medical therapy	Measure of diabetes	60	1	150	USA
10b[14]	Kheniser (2017)	Bypass	Sleeve	-	Measure of anaemia	48	1	100	USA
10c[15]	Zhou (2019)	Bypass	Sleeve	-	Measure of diabetes	60	1	100	USA
10d[16]	Lorkowski (2020)	Bypass	Sleeve	Medical therapy	Measure of cholesterol	60	1	90	USA
11	Capristo (2018)	Bypass	Sleeve	-	Measure of diabetes	12	1	120	Italy
12a	Salminen (2018)	Bypass	Sleeve	-	Measure of weight loss	60	3	240	Finland
12b	Rebelos (2019)	Bypass	Sleeve	-	Measure of brain glucose uptake	6	1	20	Finland
12c	Gronroos (2021)	Bypass	Sleeve	-	Measure of weight loss	84	3	240	Finland

13a	Hofsø (2020)	Bypass	Sleeve	-	Measure of diabetes	12	1	109	Norway
13b	Hofsø (2021)	Bypass	Sleeve	-	Measure of bone density	12	1	109	Norway
14a	Pajecki (2020)	Bypass	Sleeve	-	Measure of morbidity	3	1	36	Brazil
14b	Pajecki (2020)	Bypass	Sleeve	-	Measure of weight loss	12	1	36	Brazil
15	Wallenius (2020)	Bypass	Sleeve	-	Measure of diabetes	24	4	60	Sweden

Abbreviations: GCP – greater curvature plication; HRQL- health-related quality of life; NAFLD – non-alcoholic fatty liver disease

[1] If primary outcome was not specified, the outcome i) used to calculate sample size or ii) the first outcome listed in the abstract is given. NB. For ancillary and sub-studies this will differ from the original trial and will be the main outcome reported

[2] Number of patients randomised in total. NB. For ancillary and sub-studies this will be the number of patients randomised to the groups analysed which may be different to the total number randomised in the trial

[3] Trial registry entry states that the target sample size was n=200 with a primary outcome of weight loss at 5 years

[4] Reporting 1-year follow-up of primary outcome

[5] Reporting 3-year follow-up of primary outcome

[6] Reporting 5-year follow-up of primary outcome

[7] Reporting 1-year follow-up of primary and secondary outcomes

[8] Possible ancillary study of De Barros[3] (#5b) – analysis of additional outcomes that are not mentioned in the trials registry database. Both papers cite the same clinical trials registry number, but it is unclear as to whether the participants are the same.

[9] Article states that the primary outcome is weight loss but focusses on secondary outcomes.

[10] Trial registry entry states that the target sample size was n=620 with a primary outcome of weight loss at 5 years. Authors acknowledge poor enrolment and hence change of primary outcome

[11] Reporting primary outcome (weight loss) and secondary outcomes. In comparison to the earlier study[4], this study recruited patients from two centres and exceeded the target sample size of 620

[12] Ancillary studies of Casajoana[5] – analysis of additional outcomes not stated in the original publication or trials registry database

[13] Reporting additional primary outcomes stated in the trials registry database

[14] Ancillary study of Schauer [6] - retrospective sub-analysis of gastric bypass and sleeve trial groups (not medical therapy) data of additional outcomes not specified in the trials registry

[15] Ancillary study of Schauer [6] – analysis of gastric bypass and sleeve trial groups (not medical therapy)

[16] Ancillary study of Schauer [6] – analysis of gastric bypass, sleeve trial and medical therapy trial groups

Supplementary Table 5. Mean domain scores for PRECIS-2 assessments

	PRECIS-2 domains									
**First author**	**Eligibility**	**Recruitment**	**Setting**	**Organisation**	**Flexibility: delivery**	**Flexibility: adherence**	**Follow-up**	**Primary outcome**	**Primary analyses**	**Mean score (trial)**
Keidar	3	5	2	5	3	ND	3	3	5	3.6
Peterli	5	5	5	4	2	ND	3	5	5	4.3
Zhang	3	5	2	ND	4	ND	3	5	5	3.9
Ignat	4	5	2	ND	5	ND	3	5	ND	4.0
De Barros	5	5	2	ND	2	ND	2	4	ND	3.3
Kalinowski	5	5	2	ND	2	ND	3	5	ND	3.7
Biter	5	5	3	4	2	ND	2	5	Unclear[1]	3.7
Casajoana	3	5	2	ND	3	ND	3	2	ND	3.0
Nguyen	5	5	2	ND	4	ND	3	5	Unclear[1]	4.0
Schauer	2	4	2	ND	3	ND	4	4	5	3.4
Capristo	3	5	2	ND	5	ND	2	3	5	3.6
Salminen	5	ND	5	5	2	ND	2	5	5	4.1

Hofso	3	5	2	5	2	ND	3	4	5	3.6
Pajecki	3	ND	2	5	3	ND	4	5	ND	3.6
Wallenius	3	5	5	ND	4	ND	4	4	Unclear[1]	4.2
**Mean score (domain)**	3.7	4.9	2.7	4.7	3.1	ND	2.9	4.3	5.0	

[1] Intention-to-treat analysis was reported in the manuscript or protocol, but CONSORT flow charts and data tables suggest that some patients were excluded from analyses

Pragmatic

Equally pragmatic and explanatory

Explanatory

Supplementary Table 6. Verbatim text used to support risk of bias (ROB) judgment using the RoB 2 tool[2] for included studies

Author	ROB domain	RoB judgment	Text to support judgment	
**Keidar**	*Randomisation process*	Some concerns	From paper	"Randomisation was performed using online randomisation software...<br><br>Allocation to treatments was not concealed and patients knew which procedure they were to undergo."
			To note	[It is unclear if the reference to allocation concealment is about patient blinding or if patients were told their allocation before they consented to the trial] (KAC)
	Deviations from intended interventions	Low risk	From paper	"Allocation to treatments was not concealed and patients knew which procedure they were to undergo."
			To note	[Given the nature of the intervention, the surgeons would have known the allocation. There was no information about carer blinding] (KAC)
	Missing outcome data	Low risk	From paper	Table 1 shows a complete dataset
			To note	NA
	Measurement of the outcome	Low risk	From paper	NA
			To note	[It is unlikely that assessment of the outcome, a biochemical marker, could have been influenced by the laboratory technician's knowledge of the intervention received] (KAC)
	Selection of the reported result	Some concerns	From paper	NA

			To note	[There was no protocol or clinical trial registry entry to indicate the intended primary outcome or statistical plan. Hb1AC levels would not have been selected from multiple outcome measurements or multiple analyses] (KAC)
	Overall risk of bias	Some concerns		
**Peterli**	*Randomisation process*	Some concerns	From paper	"A central, computer-based block randomization (block size of 20) with sealed envelopes was carried out".<br><br>"If he/she agrees and has signed the informed consent, randomization is performed immediately by closed envelope, and the drawn operation type explained to him/her in detail again. The patient is given time to re-consider and the possibility not to participate or to come back a second time."
			To note	[Whilst the randomisation process seems ok, there is a concern that, after the patients have consented, they may be given time to reconsider and withdraw (which is fine) or come back a second time. This would suggest that patients can turn down the intervention if it wasn't the one they wanted with the hope of getting the one they want, the second time round. The baseline data shows that there were no differences between the two groups] (KAC)
	Deviations from intended interventions	Low risk	From paper	"There was no blinding with regard to the type of operation: patients aswell as physicians and dietitians assessing follow-up data were informed about the procedure performed."<br><br>"Analysis was performed on the intention-to-treat population."
			To note	NA (KAC)
	Missing outcome data	Low risk	From paper	"There were some missing data for weight during the study (including 12 patients that were lost to follow-up until 5 years postoperatively): 0% at baseline, 0.46% at one year, 25.8 at two years, 3.2% at three years, 31.8% at four years, and 5.5% (representing the drop-outs) at five years."<br><br>"Missing follow-up data were imputed by a multiple imputation technique using the fully conditional specification method based on Markov chain Monte Carlo simulation"
			To note	[There was a discrepancy between the completeness of the data in the main paper and that in the supplementary information from which the above quote is from. However, they reported that they used multiple imputation technique] (KAC)
	Measurement of the outcome	Low risk	From paper	"There was no blinding with regard to the type of operation: patients aswell as physicians and dietitians assessing follow-up data were informed about the procedure performed."

			To note	[Patients were unblinded, but it is unlikely that they would have altered their food consumption to purposely influence a difference between the two interventions] (KAC)
	Selection of the reported result	Low risk	From paper	NA
			To note	[Protocol was published before the results paper. Statistical plan was present but in little detail] (KAC)
	Overall risk of bias	Some concerns		
**Zhang**	*Randomisation process*	Low risk	From paper	"…Computer-generated random numbers were used to allocate the type of procedure (LRYGB or LSG), which was written on a card and sealed in a completely opaque envelope."<br><br>"Both groups were comparable in gender, age, and BMI (P>0.05)."
			To note	NA (KAC)
	Deviations from intended interventions	Low risk	From paper	"At the last year, six LSG and four LRYGB patients were lost to follow-up, but they were still evaluated in our study to obtain the intent-to-treat analysis."
			To note	[There was no information about blinding of patients, carers or people delivering the intervention, though due to the nature of the procedure, patients and surgeons were likely to have been aware of intervention allocation.] (KAC)
	Missing outcome data	Low risk	From paper	"" If subjects did not attend follow-up, they were contacted via a telephone. Each patient was questioned about weight evolution, discomfort or complications, and resolution or improvement of comorbid conditions."<br><br>"Few patients could attend clinic visit every time due to regional or financial factor, and the rate of in person follow-up was 78.1 % at 1 year, 67.8 % at 2 years, 55.7 % at 3 years, 44.8 % at 4 years, and 37.0 % at 5 years. "<br><br>NOTE: Whilst there were low numbers coming into clinic for FU, the telephone calls were ascertaining weight. Obviously, the participants could lie if they had put on weight, but this is an unknown.
			To note	[Whilst there were low numbers coming into clinic for follow-up, the telephone calls were obtaining primary outcome data] (KAC)

	Measurement of the outcome	Some concerns	From paper	NA
			To note	[The number of participants attending follow-up in clinic was quite low over the years, however participants were followed up with phone calls and their weight given over the phone. In this instance, the participants were the outcome assessors. Participants were therefore able to influence the primary outcome – though it is unlikely that the outcome was influenced by knowledge of the intervention] (KAC)
	Selection of the reported result	Some concerns	From paper	NA
			To note	[There was no protocol or clinical trial registry entry to indicate the intended primary outcome or statistical plan.] (KAC)
	Overall risk of bias	Some concerns		
**Ignat**	*Randomisation process*	Some concerns	From paper	"The randomization was done using closed envelopes. For the 100 patients required, 120 envelopes were prepared, because of an estimated minimum 10 per cent failure rate after randomization (as a consequence of insurance reimbursement practices)."<br><br>“There were no significant differences in baseline characteristics, such as preoperative age, sex distribution, weight, BMI and QoL scores between groups”
			To note	[There is no mention as to how the random sequence was generated and if allocation concealment was maintained until after the participant had consented] (KAC)
	Deviations from intended interventions	Some concerns	From paper	Figure 1. CONSORT flow diagram shows that 5 patients randomised to RYGB were refused coverage by health insurance
			To note	[There was no information about blinding of patients, carers or people delivering the intervention, though due to the nature of the procedure, patients and surgeons were likely to have been aware of intervention allocation. There was also no information about intention-to-treat analysis though Figure suggests ITT was used, despite the loss to follow-up] (KAC)
	Missing outcome data	High risk	From paper	Figure 1. CONSORT flow diagram shows that at the primary outcome follow-up time point of 3 years, 48 participants (22 RYGB and 26 sleeve) (out of 100 that received intervention) were lost to follow-up.
			To note	[There was no information about imputed data or sensitivity analyses or correction of baises] (KAC)

	Measurement of the outcome	Low risk	From paper	NA
			To note	[There was no information about blinding of the outcome assessors. It is unlikely that the outcome would have been influenced by knowledge of the intervention as the outcome is not subjective] (KAC)
	Selection of the reported result	Some concerns	From paper	NA
			To note	[There was no protocol to indicate the intended primary outcome or statistical plan] (KAC)
	Overall risk of bias	High risk		
**deBarros**	*Randomisation process*	High risk	From paper	"All of the patients were from the public health system. The patients had a "key number" through which they were registered in a local, state-run regulatory system. Since this registration was online, the computer system periodically sent a "key number" automatically to the hospital and this number was used to call in the corresponding patient. Once the invitation to participate in the research was accepted, the secretary of the department drew a number: 1 for RYGB Group or number 2 for SG Group. This approach ensured random allocation of the patients to the groups."
			To note	[The randomisation method is unconventional, and it is unclear as to whether or not it is random. There was also concern about allocation concealment and that patients could be encouraged (or discouraged) to take part in the trial] (KAC)
	Deviations from intended interventions	Some concerns	From paper	NA
			To note	[There was no information about blinding of patients, carers or people delivering the intervention. There was also no information about intention-to-treat analysis, though Figure 1 suggests that only those followed-up were analysed] (KAC)
	Missing outcome data	Low risk	From paper	Figure 1. CONSORT flow diagram shows that 3 people were lost to follow-up
			To note	[Figure 1 suggests minimal loss (3/53) to follow-up] (KAC)
	Measurement of the outcome	Low risk	From paper	"The patients underwent THE with FibroScan device in accordance with the manufacturer's instructions and training. The operators were trained and have performed more than 50 exams with this new approach in patients with obesity using the XL probe"

				“The operator was blinded with reference to the technique done in any given patient”
			To note	NA
	Selection of the reported result	Some concerns	From paper	NA
			To note	[There was no protocol, so not statistical plan. Clinical trial registry entry indicated that the intended primary outcome was that reported by the paper] (KAC)
	Overall risk of bias	Some concerns		
**Kalinowski**	*Randomisation process*	Some concerns	From paper	“Simple randomization with random allocation rule was used to assign patients to the treatment groups to ensure equal sizes of groups at the end of the trial. Sequence generation was based on a random-number table.”<br><br>“The study groups had comparable baseline BMI, age, sex, and prevalence of co-morbidities”
			To note	[There is no information about allocation concealment] (KAC)
	Deviations from intended interventions	Low risk	From paper	NA
			To note	[There was no information about blinding of patients, carers or people delivering the intervention, though due to the nature of the procedure, patients and surgeons were likely to have been aware of intervention allocation. There was also no information about intention-to-treat analysis, however given there were no crossovers, they were all analysed in their allocated treatment groups] (KAC)
	Missing outcome data	Low risk	From paper	"A dropout rate of 5-10% was expected, and groups of 36 patients each were accepted."<br><br>"Follow-up rate at 12 months was 95.8% (97% for RYGB and 94% for SG patients)."
			To note	NA
	Measurement of the outcome	Low risk	From paper	NA
			To note	[There was no information about blinding of the outcome assessors. It is unlikely that the outcome would have been influenced by knowledge of the intervention as the outcome is not subjective] (KAC)

	Selection of the reported result	Some concerns	From paper	NA
			To note	[There was no protocol to indicate the intended primary outcome or statistical plan] (KAC)
	Overall risk of bias	Some concerns		
**Biter**	*Randomisation process*	Some concerns	From paper	"These patients were randomized with a 1:1 ratio between two procedure types, i.e., LRYGB versus LSG. At randomization, patients were stratified for sex, presence of T2D, and BMI > 50 kg/m2"
			To note	[No information about how the random sequence was generated or allocation concealment. The table of baseline data suggested that the groups were balanced] (KAC)
	Deviations from intended interventions	Some concerns	From paper	"Patients that were scheduled for LRYGB but underwent a LSG—or vice versa—because of technical difficulties or perceived safety were analyzed as crossovers to the other group."
			To note	[There was no information about blinding of patients, carers or people delivering the intervention, though due to the nature of the procedure, patients and surgeons were likely to have been aware of intervention allocation. There was confusion about intention-to-treat analysis. The first paper said they used ITT whereas the later paper seems to have used per protocol analysis] (KAC)
	Missing outcome data	Some concerns	From paper	"An important limitation of this study is the high lost to follow-up rate on sweet eating behavior, especially at 2 years postoperative (64.7%)."<br><br>"Therefore, we created a copy of our dataset and used single imputation to increase power and reduce potential bias. With single imputation, missing values were substituted by the median or mode"
			To note	[There was a large loss to follow-up at 2 years. Whilst imputation was carried out, there are concerns that using the median/mode from two thirds of the participants may npt be representative of the third that dropped out] (KAC)
	Measurement of the outcome	Low risk	From paper	NA
			To note	[There was no information about blinding of the outcome assessors. It is unlikely that the outcome would have been influenced by knowledge of the intervention as the outcome is not subjective] (KAC)

	Selection of the reported result	Low risk	From paper	NA
			To note	[The protocol was available. It specified the power calculations but didn't have a defined statistical plan.] (KAC)
	Overall risk of bias	Some concerns		
**Casajoana**	*Randomisation process*	Low risk	From paper	"The randomization process was performed by the statistic department using a computer software program that generated the random sequence. The allocation of patients was assigned by simple randomization 1:1:1 to undergo mRYGB, SG or GCP, using opaque sealed sequentially numbered envelopes with stratification according to baseline levels of HbA1c (greater or lower/equal to 7%). After signing informed consent, patients were allocated to a specific surgery."<br><br>Table 1 shows that most variables were similar between groups. BMI was significantly higher in the GCP group than SG and RYGB
			To note	[The differences in BMI were probably not an indicator that there was a problem with randomisation] (KAC)
	Deviations from intended interventions	Low risk	From paper	"The study was therefore not blinded and the patients, endocrinologist and surgeon were informed about the type of surgery procedure the patient had been allocated to."
			To note	[There was also no information about intention-to-treat analysis, however there were no crossovers, so patients were analysed in the group to which they were randomised] (KAC)
	Missing outcome data	Low risk	From paper	Follow-up compliance at 1 year was 97.78% (n = 44)"
			To note	NA
	Measurement of the outcome	Low risk	From paper	"GLP-1 was measured by radioimmunoassay (Millipore, Saint Charles, MO)."
			To note	[There was no information regarding whether the outcome assessor was blinded. Since the outcome is a biochemical test assessed by lab technicians, they are likely to be blinded] (KAC)
	Selection of the reported result	Some concerns	From paper	NA
			To note	[There was no protocol to indicate the intended primary outcome or statistical plan] (KAC)

	Overall risk of bias	Some concerns		
**Nguyen**	*Randomisation process*	Some concerns	From paper	"After written consent, patients were randomly assigned to laparoscopic gastric bypass or laparoscopic gastric banding by the use of sealed envelopes. The randomized assignment was discussed with the patient at the second office visit, when the patient had the right to withdraw from the study protocol"<br>"After patients gave consent, they were randomly assigned to laparoscopic gastric bypass or laparoscopic gastric banding by use of sealed envelopes with a block of 3 groups to allow for even recruitment. The randomized assignment was discussed with the patient at the second office visit, when the patient had the right to withdraw from the study protocol."
			To note	[There is no information about how the random sequence was generated] (KAC)
	Deviations from intended interventions	High risk	From paper	"The randomized assignment was discussed with the patient at the second office visit, when the patient had the right to withdraw from the study protocol."<br><br>"Data for patients who underwent their randomized assignment were analyzed on the basis of the intention-to-treat basis. Data for patients with gastric banding that were converted to another bariatric operation were analyzed within the gastric banding group. Patients who did not undergo their randomized assignment were excluded from the analysis"<br><br>53 patients (14 RYGB and 39 gastric band) didn't go through with intervention due to either wanting the other procedure or insurance wouldn't pay.
			To note	[Patients, and seemingly, an insurance company preference suggests there were some problems with randomisation] (KAC)
	Missing outcome data	Low risk	From paper	NA
			To note	[Missing data was mainly due to withdrawals after randomisation due to health insurance not covering treatment and patients' preference. It was unlikely due to the true value] (KAC)
	Measurement of the outcome	Some concerns	From paper	NA
			To note	[There was no information about blinding of the outcome assessors. It is unlikely that the outcome would have been influenced by knowledge of the intervention as the outcome is not subjective] (KAC)

	Selection of the reported result	Some concerns	From paper	NA
			To note	[There was no protocol to indicate the intended primary outcome or statistical plan] (KAC)
	Overall risk of bias	High risk		
**Schauer**	*Randomisation process*	Low risk	From paper	"Patients will be randomized in a 1:1:1 ratio into one of the 3 treatment options. The randomization scheme will be developed by a statistician at the Cleveland Clinic Coordinating Center for Clinical Research C5Research). A blocking sequence will be used, and subjects will be stratified according to diabetes severity, defined as insulin-requiring at the time of screening. Sealed and sequentially numbered envelopes containing the treatment assignment will be provided to the nurse coordinator responsible for patient enrollment. Once eligibility is confirmed, the nurse will select and open the next sealed envelope in sequence to reveal the treatment assignment. Envelopes must be selected in sequence and not to be opened prior randomization."
			To note	[Protocol states informed consent is taken at visit 1/screening and randomisation occurs at baseline/visit 2] (KAC)
	Deviations from intended interventions	Low risk	From paper	"Patients and study personnel will not be blinded to treatment assignment. The treatment assignment will remain unknown until the patient is randomized after meeting all eligibility requirements."<br><br>"…modified ITT to be used"
			To note	NA
	Missing outcome data	Low risk	From paper	NA
			To note	[Only one patient dropped out} (KAC)
	Measurement of the outcome	Low risk	From paper	NA
			To note	[There was no information regarding whether the outcome assessor was blinded. Since the outcome is a biochemical test assessed by lab technicians, they are likely to be blinded] (KAC)
	Selection of the reported result	Some concerns	From paper	NA

			To note	[Protocol was available but was published at the same time as the first result paper. It is unclear if the protocol has been adapted to fit the results] (KAC)
	Overall risk of bias	Some concerns		
**Capristo**	*Randomisation process*	Some concerns	From paper	"A total of 120 patients were enrolled and randomly assigned 1:1 to RYGB or to SG"<br><br>"Between-groups baseline variables (Table 1) as well as sex (33.3% men and 66.7% women) did not differ significantly between treatment groups (P = 0.133)."
			To note	[There is no information about how the random sequence was generated or any information about allocation concealment] (KAC)
	Deviations from intended interventions	Low risk	From paper	"A single centre, open, 1-year randomised trial to compare the incidence of hypoglycaemia after RYGB or SG."<br><br>"Three patients withdrew their consent before the first visit after surgery and were excluded from the analysis. Two of the 3 patients who withdrew their consent in the SG arm after the visit at 6 months and 2 patients in the RYGB arm who withdrew after the visit at 3 months did so because of job-related changes in location. One patient withdrew consent in the SG arm after the 6-month visit did because of family problems."<br><br>"These patients were included in the intention-to-treat analysis."
			To note	NA
	Missing outcome data	Low risk	From paper	NA
			To note	[Data suggested that there was minimal missing data] (KAC)
	Measurement of the outcome	Low risk	From paper	"A single centre, open, 1-year randomised trial to compare the incidence of hypoglycaemia after RYGB or SG."
			To note	[Trial described as an open trial so outcome assessors may have been unblinded. OGTT a difficult test to administer due to discomfort. Unsure if test would be more difficult for either group] (KAC)
	Selection of the reported result	Low risk	From paper	NA
			To note	[Protocol was published before the results paper. Statistical plan in line with what was presented] (KAC)

	Overall risk of bias	Some concerns		
**Salminen**	*Randomisation process*	Low risk	From paper	"Randomization was performed with a 1:1 equal allocation ratio. The opaque, sealed, and sequentially numbered randomization envelopes were shuffled and then distributed to each participating hospital. To randomize an eligible patient after the clinical decision of proceeding to bariatric surgery for treating morbid obesity, the surgeon opened a sealed envelope containing the information of the assigned randomization group. All of the treating surgeons were part of the study team"<br><br>"…there were no differences in demographic characteristics between the study groups regarding patient age, sex, BMI, and obesity-associated comorbidities"
			To note	NA
	Deviations from intended interventions	Low risk	From paper	"Analyses were performed according to the intention-to-treat population, ie, all patients were analyzed in their original intervention group, and missing data were excluded from the analyses. Because of missing values at at least 1 time point (60/240 patients [25%]), a sensitivity analysis using multiple imputation was performed for the primary outcome (percentage excess weight loss). Multivariate imputation by fully conditional specification method was performed"
			To note	[Trial was described as open label] (KAC)
	Missing outcome data	Low risk	From paper	"However, the drop-out rates were similar in both groups. Multiple-imputation analysis suggested that there was little risk for bias when percentage excess weight loss was compared between the 2 procedures"
			To note	NA
	Measurement of the outcome	Low risk	From paper	"Patients were evaluated at outpatient control visits, and all prespecified data were thoroughly recorded."
			To note	[Trial was described as open label so outcome assessors were unlikely blinded. It is unlikely that the outcome would have been influenced by knowledge of the intervention as the outcome is not subjective.]
	Selection of the reported result	Low risk	From paper	NA
			To note	[The protocol was available. The statistical plan lacked detail but it was sufficient] (KAC)

	Overall risk of bias	Low risk		
**Hofsø**	*Randomisation process*	Low risk	From paper	"Randomisation and masking Patients were randomly allocated (1:1) to gastric bypass or sleeve gastrectomy with use of a computerised random number generator with a block size of 10. The surgeon generating the randomisation sequence (MS) was not involved in patient follow-up. Sequentially numbered, sealed opaque envelopes were used to conceal allocation, and the allocation was revealed in the operating theatre by the bariatric surgeon on the day of surgery"<br><br>"Patient characteristics were similar between groups"
			To note	NA
	Deviations from intended interventions	Low risk	From paper	"The Oseberg study is a single-centre, triple-blind, randomised controlled trial. All study personnel, patients, and the primary outcome assessor (MCS) were blinded to allocations. The surgeons used identical skin incisions during both surgeries and did not participate in patient follow-up."
			To note	[The paper states it used ITT and PP analysis] (KAC)
	Missing outcome data	Low risk	From paper	Figure 1 shows the number of participants attending follow-up
			To note	[One participant from each group dropped out but were included in analyses]
	Measurement of the outcome	Low risk	From paper	"Whole-blood HbA1c was analysed on a Tosoh high-performance liquid chromatography G8 analyser (Tosoh Corporation, Tokyo, Japan)."
			To note	[Primary outcome is a standard biochemical test. Assessors blinded] (KAC)
	Selection of the reported result	Low risk	From paper	"Primary outcomes Remission of type 2 diabetes Whole blood HbA1c will be analysed on a Tosoh high-performance liquid chromatography G8 analyser (Tosoh Corporation, Tokyo, Japan) with reagents from the supplier"
			To note	[Protocol available and published before the results paper. Assessment completed as stated in protocol] (KAC)
	Overall risk of bias	Low risk		
**Pajecki**	*Randomisation process*	Low risk	From paper	"Patients were assigned to either group using a computer-based randomization with sealed envelopes after consent was obtained"

				“The 2 groups were similar in terms of age, weight, and co-morbidities, but LSG patients were all female (P<.01)”
			To note	NA
	Deviations from intended interventions	Low risk	From paper	NA
			To note	[There was also no information about blinding or intention-to-treat analysis, however there were no crossovers, so patients were analysed in the group to which they were randomised] (KAC)
	Missing outcome data	Low risk	From paper	“Nine patients previously recruited for the study declined participation and even surgical treatment outside the study protocol, mostly due to family concerns regarding age and surgical outcome.”
			To note	[Unclear if the nine that withdrew were randomised. They withdrew before surgery, so the value of the outcome is irrelevant] (KAC)
	Measurement of the outcome	Low risk	From paper	“As limitations of this trial, the relatively small sample size, the short follow-up (12 months), and the single-center and open-label nature of the study may reduce evidence strength.”
			To note	[Trial described as open-label, so outcome assessors likely to be unblinded. It is unlikely that the outcome would have been influenced by knowledge of the intervention as the outcome is not subjective] (KAC)
	Selection of the reported result	Some concerns	From paper	NA
			To note	[There was no protocol to indicate the intended primary outcome or statistical plan] (KAC)
	Overall risk of bias	Some concerns		
**Wallenius**	*Randomisation process*	Low risk	From paper	“After signing informed consent patients were randomized by the use of computer-generated numbers into SG or RYGB groups.”<br><br>“There were no differences at baseline between the groups regarding age, sex, BMI, waist circumference, diabetes duration, and co-morbidities (Table 1).”
			To note	NA
	Deviations from intended interventions	Some concerns	From paper	“One patient randomised to RYGB wanted SG so was excluded.”

				"Analysis was performed in accordance with intention to treat"
			To note	[Clinical trials website suggested it was an open-label trial] (KAC)
	Missing outcome data	Low risk	From paper	Fig 1 shows that 11/60 randomised did not receive allocated surgery and were excluded. Of those that received the intervention, all were followed-up at the 12-month data collection point.
			To note	NA
	Measurement of the outcome	Low risk	From paper	NA
			To note	[Trial described as open-label, so outcome assessors likely to be unblinded. Primary outcome is a standard biochemical test so it is unlikely that the outcome would have been influenced by knowledge of the intervention (KAC)
	Selection of the reported result	Some concerns	From paper	NA
			To note	[There was no protocol to indicate the intended primary outcome or statistical plan] (KAC)
	Overall risk of bias	Some concerns		

References

1. Loudon K, Treweek S, Sullivan F, et al. The PRECIS-2 tool: designing trials that are fit for purpose. *BMJ* 2015; 350:h2147.
2. Sterne JAC, Savović J, Page MJ, et al. RoB 2: a revised tool for assessing risk of bias in randomised trials. *BMJ* 2019; 366:l4898.
3. de Barros F, Fonseca ABM. Bariatric surgery during the evolution of fatty liver-A randomized clinical trial comparing gastric bypass and sleeve gastrectomy based on transient elastography. *Clinical Obesity* 2020.
4. Biter LU, van Buuren MMA, Mannaerts GHH, et al. Quality of Life 1 Year After Laparoscopic Sleeve Gastrectomy Versus Laparoscopic Roux-en-Y Gastric Bypass: a Randomized Controlled Trial Focusing on Gastroesophageal Reflux Disease. *Obes Surg* 2017; 27(10):2557-2565.
5. Casajoana A, Pujol J, Garcia A, et al. Predictive Value of Gut Peptides in T2D Remission: Randomized Controlled Trial Comparing Metabolic Gastric Bypass, Sleeve Gastrectomy and Greater Curvature Plication. *Obes Surg* 2017; 27(9):2235-2245.
6. Schauer PR, Bhatt DL, Kirwan JP, et al. Bariatric Surgery versus Intensive Medical Therapy for Diabetes - 5-Year Outcomes. *The New England journal of medicine* 2017; 376(7):641-651.
